# Novel magnetic resonance wave intensity analysis in pulmonary hypertension

**DOI:** 10.1186/1532-429X-16-S1-P252

**Published:** 2014-01-16

**Authors:** Michael A Quail, Daniel S Knight, Jennifer A Steeden, Andrew Taylor, Vivek Muthurangu

**Affiliations:** 1 Centre for Cardiovascular Imaging, Institute of Cardiovascular Science, London, UK; 2 Great Ormond Street Hospital for Children, London, UK

## Background

In pulmonary arterial hypertension (PH), abnormal wave reflections play an important part in pathophysiology and can be assessed using wave intensity analysis (WIA). However, conventionally this technique requires simultaneous invasive measurement of pulmonary artery pressure and velocity. Therefore, we have developed a novel non-invasive technique that uses high temporal-resolution phase-contrast MR (PCMR) flow and area data to perform WIA. The aim of this study was to establish any differences in wave reflections between patients with PH and healthy volunteers.

## Methods

Right PA volume flow and area curves were obtained in 15 patients with PH (mean ± SD, age 52 ± 13 years) and 10 healthy controls (age 45 ± 11 years) using a retrospectively gated, respiratory navigated, golden-angle, high TR, PCMR sequence. The right PA was used to avoid the through plane motion in the main PA. All patients also underwent right heart cardiac catheterization for pressure and vascular resistance (PVR) measurement within 30days (mean 11days) and had serum brain natriuretic peptide (BNP) measured. Wave speed was determined in the right PA using the single slice Q-A method. WIA was derived in terms of volume flow and area changes.

## Results

**Figure 1:**
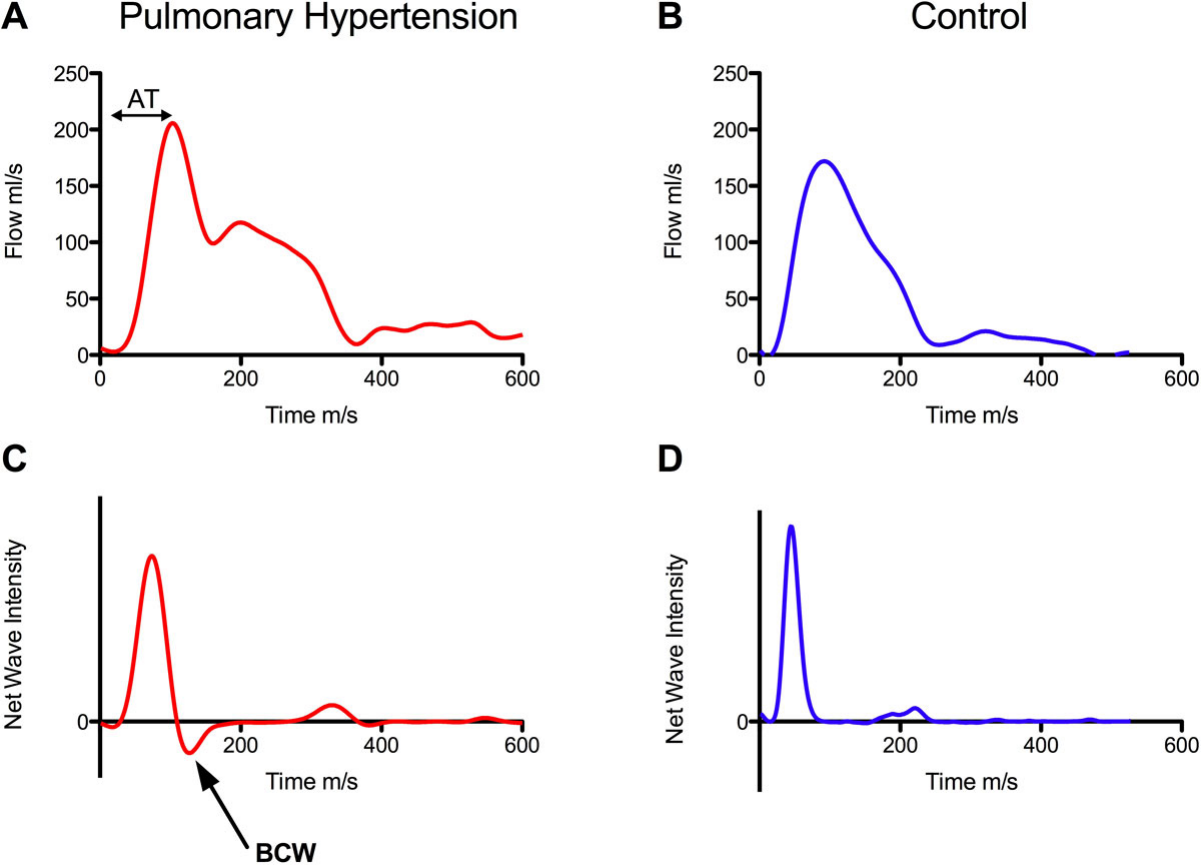
**Example flow curves from a PH patient (A) and a healthy control (B), demonstrating abnormal contour in PH**. Net wave intensity plots from PH (C) and healthy control (D) showing the presence of a backwards compression wave (BCW). AT, Acceleration time.

There were significant differences in WIA between cases and controls (Table 1 Figure 1). Wave speed was higher in PH than controls in keeping with reduced arterial compliance (p = 0.0001). A backwards compression wave (BCW) was observed in all patients with PH (Figure 1C), but was absent in all control patients (p < 0.0001). Conversely a backwards expansion wave was seen in normal controls but not in PH. Average PVR and PA mean arterial pressure (MAP) were 612 ± 298 ARU and 43 ± 12 mmHg respectively. There was a significant correlation between MAP and the duration of the BCW (R = 0.62, p = 0.01) and also the ratio of the magnitude of the forward (FCW) and backwards compression waves (R = -0.57, p = 0.03). PVR was independently associated with the acceleration time (AT, Figure 1A) (β = -1.42, p = 0.005) and the time of onset of the BCW (β = 0.95, p = 0.039) by multiple linear regression analysis (Model, R = 0.74). The ratio of the FCW and BCW correlated significantly with serum BNP (R = 0.63, p = 0.017).

**Table 1:** Data Table, Pulmonary Hypertension and Healthy Controls

	Pulmonary Hypertension n = 15	Control n = 10	probablity
Age (years)	52 ± 13	45 ± 12	NS

Wave Speed (m/s)	1.2 ± 0.3	0.7 ± 0.2	p = 0.0001

Backwards Compression Wave (BCW)	15/15	0/10	p < 1e^-6^

Backwards Expansion Wave (BEW)	0/15	10/10	p < 1e^-6^

Acceleration Time (ms)	60 ± 19	108 ± 22	P < 7e^-5^

All values expressed mean ± standard deviation (SD), NS= non-significant.

## Conclusions

We have demonstrated that it is possible to assess abnormal hemodynamic abnormalities in PH using CMR based WIA. Specifically, patients with PH have an abnormal backwards-traveling compression wave, which probably arises from the narrowed distal vasculature, augmenting pressure and reducing flow. Furthermore, there is also loss of the normal backwards expansion wave that is thought to increase flow in the pulmonary artery. Importantly, we have shown that WIA indices correlate strongly with pressure, vascular resistance and serum markers of disease severity, and therefore show promise as a diagnostic tool for PH.

## Funding

British Heart Foundation.

